# HEATHER BERND CLARKE, MBA, LCSW, PA HEALTH & WELLNESS, MECHANICSBURG, PA, UNITED STATES

**DOI:** 10.1093/geroni/igae098.1347

**Published:** 2024-12-31

**Authors:** Heather Clarke

**Affiliations:** PA Health & Wellness, Pittsburgh, Pennsylvania, United States

## Abstract

PA Health & Wellness, a managed care organization in Pennsylvania, has collaborated with Johns Hopkins on deploying the academic Maximizing Independence at Home (“MIND at Home”) Program for community-living individuals with cognitive impairment who have Medicaid Long-Term Support Services. This home-based model is aimed at coordinating care and supporting caregivers for individuals who are cognitively complex and at risk for out of home placement. The model consists of a non-clinical Memory Care Coordinator (MCCs) who is supported by a Memory Care Manager RN to provide a holistic approach to assisting the individual remain in the community and decrease caregiver burnout. This is accomplished through a specialized assessment of 13-memory care domains that drive the development of an individualized care plan. Implementation of the care plan by the MCC includes assistance in navigating the managed care system, coordination of provider appointments and benefits, referral to community resources, and disease education and coaching for the family caregiver. This presentation will describe the program implementation within PA Health & Wellness since 2022. We will present key components of program implementation, operations and learned experiences. We will also review preliminary results on program impact showing reductions in inpatient and emergency room visits for individuals enrolled in the program compared to similar, non-enrolled, individuals.

